# Strain-, Sex-, and Time-Dependent Antidepressant-like Effects of Cannabidiol

**DOI:** 10.3390/ph14121269

**Published:** 2021-12-06

**Authors:** Gabriela P. Silote, Michelle C. Gatto, Amanda Eskelund, Francisco S. Guimarães, Gregers Wegener, Sâmia R. L. Joca

**Affiliations:** 1 Department of Biomolecular Sciences, School of Pharmaceutical Sciences of Ribeirão Preto (FCFRP), University of São Paulo (USP), Ribeirão Preto 14040-903, SP, Brazil; gabrielasilote@gmail.com (G.P.S.); michelle.gatto@hotmail.com (M.C.G.); 2 Translational Neuropsychiatry Unit (TNU), Department of Clinical Medicine, Aarhus University, 8000 Aarhus, Denmark; ares@clin.au.dk; 3 Department of Pharmacology, School of Medicine of Ribeirão Preto, University of São Paulo, Ribeirão Preto 14049-900, SP, Brazil; fsguimar@fmrp.usp.br; 4 Center for Interdisciplinary Research on Applied Neurosciences (NAPNA), University of São Paulo, São Paulo 05508-000, SP, Brazil; 5 Department of Biomedicine, Aarhus University, 8000 Aarhus, Denmark

**Keywords:** cannabidiol, S-ketamine, sex, mice strain, Flinders Sensitive Line rats, Flinders Resistant Lines rats, tail suspension test, forced swim test

## Abstract

Cannabidiol (CBD) is a non-intoxicating compound extracted from *Cannabis sativa*, showing antidepressant-like effects in different rodent models. However, inconsistent results have been described depending on the species and the strain used to assess depressive-like behavior. Moreover, only a few studies investigated the effect of CBD in female rodents. Therefore, we aimed to (i) investigate the effects of CBD in two different strains of mice (Swiss and C57BL/6) and a rat model of depression based on selective breeding (Flinders Sensitive and Resistant Lines, FSL and FRL) subjected to tests predictive of antidepressant-like effects and (ii) investigate the influence of sex in the effects of CBD in both mice and rats. CBD induced an antidepressant-like effect in male Swiss but not in female Swiss or C57BL/6 mice in the tail suspension test (TST). In male FSL rats, CBD produced an antidepressant-like effect 1 h post injection. However, in female FSL, CBD induced a bimodal effect, increasing the immobility time at 1 h and decreasing it at 2 h. In conclusion, strain, sex, and administration time affect CBD’s behavioral response to rodents exposed to tests predictive of antidepressant effects.

## 1. Introduction

Major depressive disorder (MDD) is a chronic and disabling psychiatric disorder [1]. The World Health Organization (WHO) estimates that more than 300 million people suffer from depression worldwide [2,3] and that MDD is a major contributor to the world’s burden of disease [4,5]. This scenario is further aggravated by the high prevalence and comorbidity of anxiety disorders, affecting more than 3.6% of the world population [2]. Consequently, there is a significant socioeconomic impact with increasing health-related costs and a reduction in the productivity of the economically active population [6,7]. Importantly, there is a considerable sex imbalance in MDD and anxiety prevalence, with depression being twice as prevalent in women than men [2,3]. Despite that, the use of females to investigate new drugs and neuropathology is scarce in basic research [8,9,10]. Eighty percent of the preclinical research in behavioral neuroscience has been developed in male subjects [11], which can potentially limit the benefit of the discoveries for females and compromise the development of personalized medicine [8,11,12,13].

Cannabidiol (CBD) is one of the main active constituents present in *Cannabis sativa* [14]. Unlike delta-9-tetrahydrocannabinol (Δ9-THC), it does not induce psychostimulant effects, nor is it associated with an increased risk of abuse and dependence [15]. Due to its non-intoxicating properties and multitargeted action [16,17], the therapeutic properties of CBD were investigated in several animal models of neurological and psychiatric disorders, with promising results [18]. The anxiolytic effect of CBD was shown in different animal models [19,20,21] and clinical trials [19,20,21]. Although consistent data from humans are still lacking [16], the antidepressant properties of CBD were consistently demonstrated in different behavioral readouts, such as the Forced Swim Test (FST) [22,23,24,25,26], Tail Suspension Test (TST) [27], Learned Helplessness (LH) [24], Olfactory Bulbectomy (OBX) [28], and Chronic Unpredictable Mild Stress (CUMS) [29,30]. Moreover, CBD is also able to rescue the behavioral phenotype of congenitally depressed rat strains, such as the Wistar-Kyoto (WKY) [31,32] and Flinders Sensitive Line (FSL) rats [24,32]. Interestingly, CBD produces a rapid and sustained antidepressant-like effect in rodents, similar to ketamine [24]. This characteristic places CBD as an interesting new drug to successfully treat depression and anxiety.

However, most studies investigating CBD antidepressant effects were performed in male rodents [16]. More recently, Shbiro and colleagues [32] investigated the effects of CBD in both female and male WKY and FSL rats and reported that CBD induced antidepressant-like effects in male and female WKY and male FSL rats [32]. However, in this study, the authors investigated only one dose of CBD (30 mg/kg), making it difficult to conclude that CBD lacks effects in female FSL rats, since this drug is known to produce an inverted U-shape dose-response curve [24,26,33,34]. Moreover, significant variability in effective CBD doses were observed (10–200 mg/kg), depending on the rodent species and strain, treatment time, and test used (reviewed by [16]). Indeed, a growing body of evidence suggests that strain and species of the selected rodents may affect baseline behavioral measurements in distinct paradigms [35,36,37,38,39,40,41]. These factors may also influence the drug response, interfering with the effective dose range [36,37,40,42] or causing the absence of effect in different tests [37,38,43]. 

Therefore, we examined CBD anxiolytic and antidepressant-like effects in both sexes of different rodent species (rats and mice). More specifically, the present study aimed to investigate whether CBD treatment could produce (i) an anxiolytic and antidepressant-like effect in male and female Swiss and C57BL/6 mice (the two most-used mice strains) submitted to TST and elevated plus maze (EPM) and (ii) an antidepressant-like effect in male and female FSL rats exposed to FST at different time points (1 and 2 h before the test).

## 2. Results

### 2.1. Swiss and C57BL/6 Mice

#### CBD Effects in Male and Female Swiss and C57BL/6 Mice Submitted to the Elevated Plus Maze (EPM) and Tail Suspension Test (TST)

A three-way ANOVA was performed to examine the effects on mice (Swiss and C57BL/6) of treatment, sex, and strain on the parameters evaluated in the TST and EPM. There was a significant effect of the treatment (three-way ANOVA: F(4, 141) = 7.69; *p* < 0.001) and mice strain on immobility in the TST (three-way ANOVA: F(4, 141) = 289.7; *p* < 0.001). Swiss mice presented greater immobility compared to C57BL/6 mice. Moreover, there was an interaction between treatment vs. sex (F(4, 141) = 3.31; *p* = 0.013) and a statistical tendency in the interaction treatment vs. strain (F(4, 141) = 2.21; *p* = 0.07).

**Figure 1:**
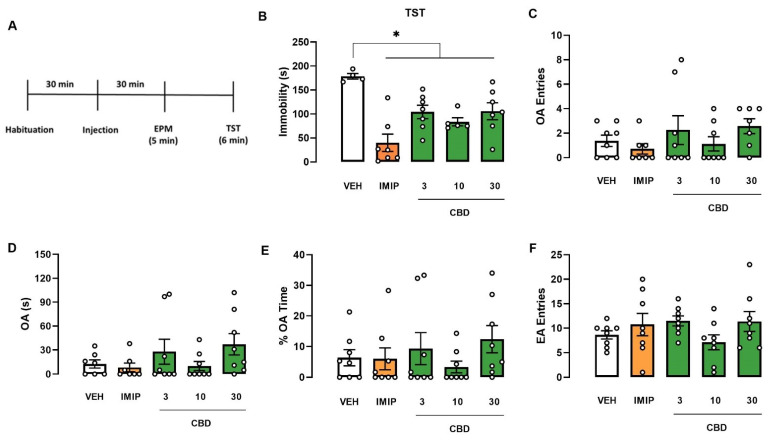
Effects induced by cannabidiol (CBD) in male Swiss mice submitted to tail suspension test and elevated plus-maze. Experimental scheme (**A**). Effect of cannabidiol (CBD) in male Swiss mice administered 30 min before the exposure to TST (**B**). EPM (**C**–**F**). Bars represent the immobility time (s) in the TST, time and percentage of the time spent on OA, number of OA and EA entries in the EPM. Values are mean  ±  SEM; asterisk represents significant treatment difference from control (* *p* < 0.05; one-way ANOVA followed by Dunnett post hoc test), n = 4–8 animals/group. CBD: cannabidiol; EA: enclosed arm; IMIP: imipramine; OA: open arm; VEH: vehicle.

**Figure 2:**
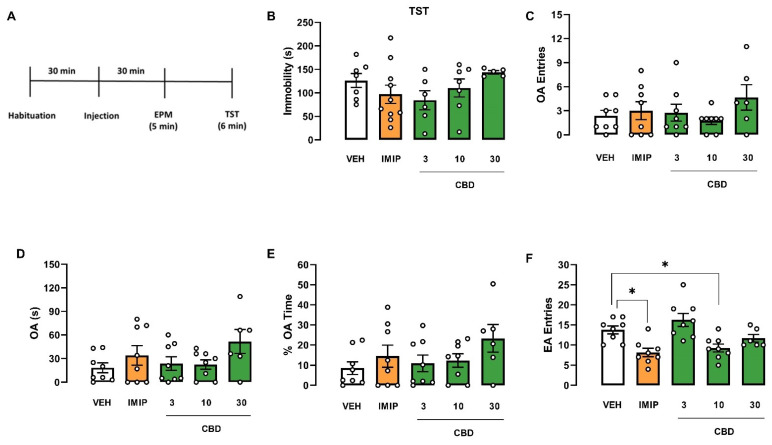
Effects induced by cannabidiol (CBD) in female Swiss mice submitted to the tail suspension test and elevated plus maze. Experimental scheme (**A**). Effect of cannabidiol (CBD) in female Swiss mice administered 30 min before the exposure to the TST (**B**) and EPM (**C**–**F**). Bars represent the immobility time (s) in the TST, time and percentage of the time spent on OA, number of OA and EA entries in the EPM. Values are mean  ±  SEM; asterisk represents significant treatment difference from control (* *p* < 0.05; one-way ANOVA followed by Dunnett post hoc test), n = 5–10 animals/group. CBD: cannabidiol; EA: enclosed arm; IMIP: imipramine; OA: open arm; VEH: vehicle.

**Figure 3:**
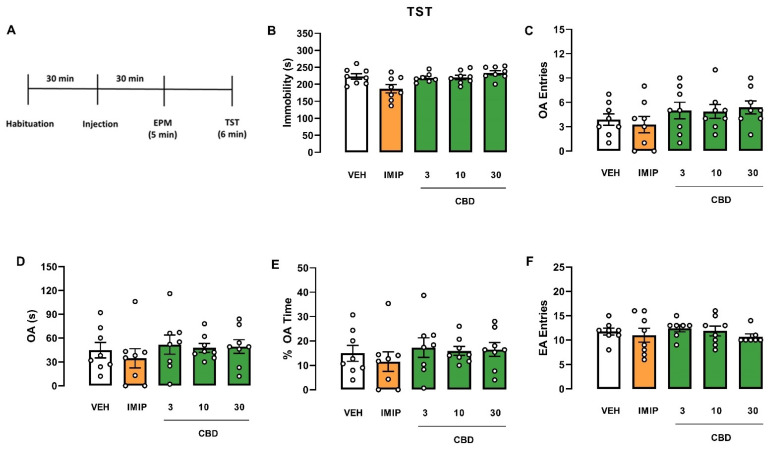
Effects induced by cannabidiol (CBD) in male C57BL/6 mice submitted to the tail suspension test and elevated plus maze. Experimental scheme (**A**). Effect of cannabidiol (CBD) in male C57BL/6 mice administered 30 min before the exposure to the TST (**B**) and EPM (**C**–**F**). Bars represent the immobility time (s) in the TST, time and percentage of the time spent on OA, number of OA and EA entries in the EPM. Values are mean  ±  SEM; n = 7–8 animals/group. CBD: cannabidiol; EA: enclosed arm; IMIP: imipramine; OA: open arm; VEH: vehicle.

**Figure 4:**
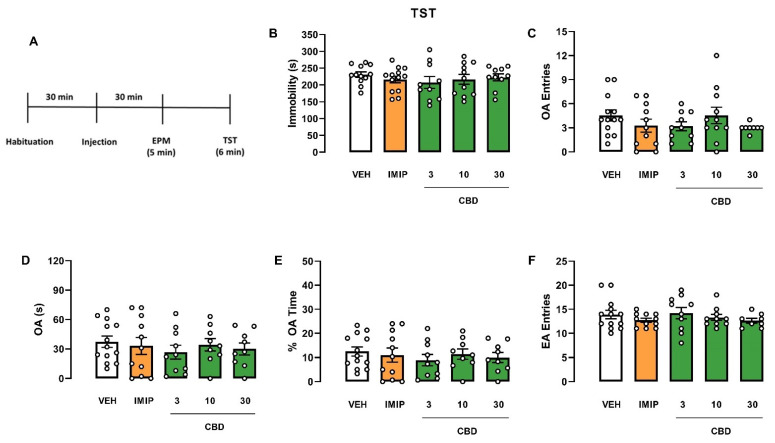
Effects induced by cannabidiol (CBD) in female C57BL/6 mice submitted to the tail suspension test and elevated plus maze. Experimental scheme (**A**). Effect of cannabidiol (CBD) in female C57BL/6 mice administered 30 min before the exposure to TST (**A**) and EPM (**B**–**F**). Bars represent the immobility time (s) in the TST, time and percentage of the time spent on OA, number of OA and EA entries in the EPM. Values are mean  ±  SEM; n = 9–13 animals/group. CBD: cannabidiol; EA: enclosed arm; IMIP: imipramine; OA: open arm; VEH: vehicle.

Since there was a significant effect on the strain and a tendency in the interaction (treatment vs. strain), we performed an independent two-way ANOVA to evaluate the treatment and sex effects in each mice strain, Swiss and C57BL/6. In Swiss mice, the two-way ANOVA revealed a significant effect of the treatment (F(4, 56) = 5.21; *p* = 0.001) and the interaction (sex vs. treatment; F(4, 56) = 2.54; *p* = 0.05). Afterwards, a one-way ANOVA was performed on each sex to compare the treatment effect on immobility. In male Swiss mice, a single injection of imipramine (IMIP) and all doses of CBD decreased immobility time in the TST (one-way ANOVA: F(4, 25) = 8657; *p* = 0.0002; Dunnett test: IMIP, *p* < 0.0001; CBD 3 mg/kg, *p* = 0.0159; CBD 10 mg/kg, *p* = 0.0038; CBD 30 mg/kg, *p* = 0.0182; Cohen test: VEH vs. IMIP: d = 3.983; VEH vs. CBD groups: f = 0.70; Figure 1B), suggesting an antidepressant-like effect. However, none of the drug treatments modified the analyzed parameters in female Swiss mice (Kruskal–Wallis test: H(5) = 6.153; *p* = 0.188; Figure 2B). A two-way ANOVA showed no effect on C57BL/6 mice (Figure 3B and Figure 4B).

Regarding the anxiety-related behavior assessed in the EPM, a three-way ANOVA revealed a significant strain effect (three-way ANOVA: F(1, 150) = 22.43; *p* < 0.001), interaction sex vs. strain (three-way ANOVA: F(1, 141) = 7.84; *p* < 0.001), and no treatment effect on the OA entries, revealing that C57BL/6 mice explored OA more than Swiss mice. The difference was more significant in male than female animals. Moreover, there was significant sex vs. strain interaction in the percentage of time spent in the OA (three-way ANOVA: F(1, 151) = 10.74; *p* = 0.001), revealing that male Swiss mice spent less time exploring the OA than their female counterparts. In C57BL/6 mice, the exploratory response was similar between sexes (Figure 1E, Figure 2E, Figure 3E, Figure 4E).

Furthermore, in the time spent in the OA, a three-way ANOVA revealed a significant difference in the sex (three-way ANOVA: F(1, 148) = 12.84; *p* < 0.001), strain (three-way ANOVA: F(1, 148) = 9.58; *p* < 0.001), and the treatment vs. strain interaction (three-way ANOVA: F(1, 148) = 2.80; *p* = 0.028), demonstrating that female mice spent more time exploring the OA compared to males. Following the significant treatment vs. strain interaction, we performed a two-way ANOVA to evaluate the treatment and strain effects in Swiss and C57BL/6 mice. There was no difference in C57BL/6 mice (Figure 3 and Figure 4). On the other hand, there was a significant treatment effect on the OA of the Swiss mice (two-way ANOVA: F(4, 73) = 4.08; *p* = 0.005). However, a one-way ANOVA or Kruskal–Wallis in each sex of the Swiss mice failed to find a significant effect in this parameter (male: Kruskal–Wallis test: H(5) = 4.792; *p* = 0.3094; Figure 1D; female: one-way ANOVA: F(4, 148) = 1.646; *p* = 0.1862; Figure 2D).

Concerning the EA entries, a three-way ANOVA revealed a significant treatment effect in this behavioral response (F(4, 148) = 5.85; *p* = 0.001). Only in female Swiss mice, imipramine and CBD 10 mg/kg treatment reduced the EA entries in the EPM compared to VEH-treated mice (one-way ANOVA: F(4, 33) = 8.305; *p* < 0.001; Dunnett test: IMIP, *p* = 0.0054; CBD 10 mg/kg, *p* = 0.0310; Figure 2F). There was no significant correlation between immobility time and EA entries in the IMIP and CBD 10 mg/kg treated group (correlation: IMIP, r = −0.5697; *p* = 0.1405; CBD10, r = 0.1224; *p* = 0.7937; data not shown). Therefore, the alteration in the locomotor activity did not affect the immobility in the TST. There was no statistical difference in male Swiss (Figure 1F) and C57BJ/6 mice of both sexes (male: Figure 3F; female: Figure 4F).

In summary, CBD induced an antidepressant-like effect in male Swiss mice without affecting the locomotor activity, but not in females from the same strain. Moreover, CBD did not affect the TST and EPM behaviors in male and female C57BL/6 mice.

### 2.2. FSL Rats

#### 2.2.1. Dose-Response Curve of Ketamine in Female FSL Rats Exposed to the OFT/FST

**Figure 5:**
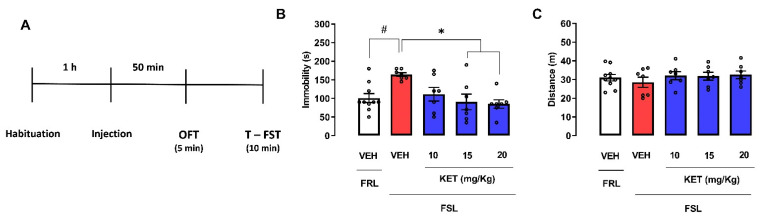
Dose-response curve of S-ketamine in female FSL rats submitted to the forced swim and open field tests. Experimental scheme (**A**). The rats were treated with VEH or ketamine (10, 15, and 20 mg/kg) 1 h before the exposition to the FST (**B**) and OFT (**C**). Bars represent the immobility time (s) in the FST or the traveled distance (m) in the OFT. Values are mean  ±  SEM; hash indicates significant differences between FSL and FRL vehicle-treated groups (# *p* < 0.05, Student’s *t*-test or Mann–Whitney test); asterisk represents significant treatment difference from FSL control (* *p* < 0.05; Kruskal–Wallis followed by Dunn’s post hoc), n = 7–10 animals/group. FST: forced swimming test; OFT: open field test; KET: S-ketamine; VEH: vehicle.

As expected, ketamine 15 mg/kg and 20 mg/kg reduced the immobility in FSL rats exposed to the FST (Kruskal–Wallis test: H(4) = 10.60; *p* = 0.0141; Dunn’s and Cohen d tests: ketamine 15 mg/kg, *p* = 0.0198, d = 1.838; ketamine 20 mg/kg, *p* = 0.0108, d = 3.350; Figure 5B). FSL rats treated with vehicle had significantly higher immobility time when compared with FRL rats treated with vehicle (Mann–Whitney test: U = 6.5; *p* = 0.0037; Figure 5B), which characterized a depressive-like phenotype. Neither rat strain (Student’s *t*-test: t(15) = 0.7981; *p* = 0.4373) nor drug treatment (one-way ANOVA: F(3, 24) = 0.6495; *p* = 0.5910; Figure 5C) changed the OFT locomotor activity in FSL rats.

#### 2.2.2. Effect Produced by CBD Administered 1 or 2 h before the OFT/FST in Male and Female FSL Rats

A three-way ANOVA was performed to examine the effect of treatment, sex, and time in the parameters evaluated in the FST and OFT. There was a significant effect of treatment (three-way ANOVA: F(4, 94) = 10.29; *p* < 0.001) and sex (three-way ANOVA: F(1, 94) = 25.48; *p* < 0.001) and between treatment vs. time (three-way ANOVA: F(2, 94) = 3.82; *p* = 0.025), sex vs. time (three-way ANOVA: F(1, 94) = 28.53; *p* < 0.001), and treatment vs. sex vs. time (three-way ANOVA: F(2, 94) = 15.41; *p* < 0.001) interactions in the immobility in the FST.

Furthermore, in the total distance traveled in the OFT, there was a significant effect of treatment (three-way ANOVA: F(4, 94) = 12.80; *p* < 0.001), sex (three-way ANOVA: F(1, 94) = 38.16; *p* < 0.001), and time (three-way ANOVA: F(1, 94) = 7.32; *p* < 0.001) and treatment vs. sex (three-way ANOVA: F(4, 94) = 13.58; *p* < 0.001), treatment vs. time (three-way ANOVA: F(2, 94) = 3.96; *p* = 0.022), sex vs. time (three-way ANOVA: F(4, 94) = 4.27; *p* = 0.04), and treatment vs. sex vs. time (three-way ANOVA: F(2, 94) = 7.46; *p* = 0.0001) interactions. 

Since there was a significant effect in the interaction (treatment vs. sex vs. time) in the parameters assessed in FST and OFT, we performed an independent Student’s *t*-test to compare the results between the rat strains, FRL and FSL, vehicle-treated groups, and a one-way ANOVA followed by Dunnett post hoc test to evaluate the treatment effect in each sex and time in FSL rats. When the variances between the groups were not homogenous, the Mann–Whitney (for comparisons between FSL and FRL vehicle-treated groups) or Kruskal–Wallis followed by Dunn’s post hoc tests were used to compare FSL rats treated with VEH, ketamine, or CBD.

**Figure 6:**
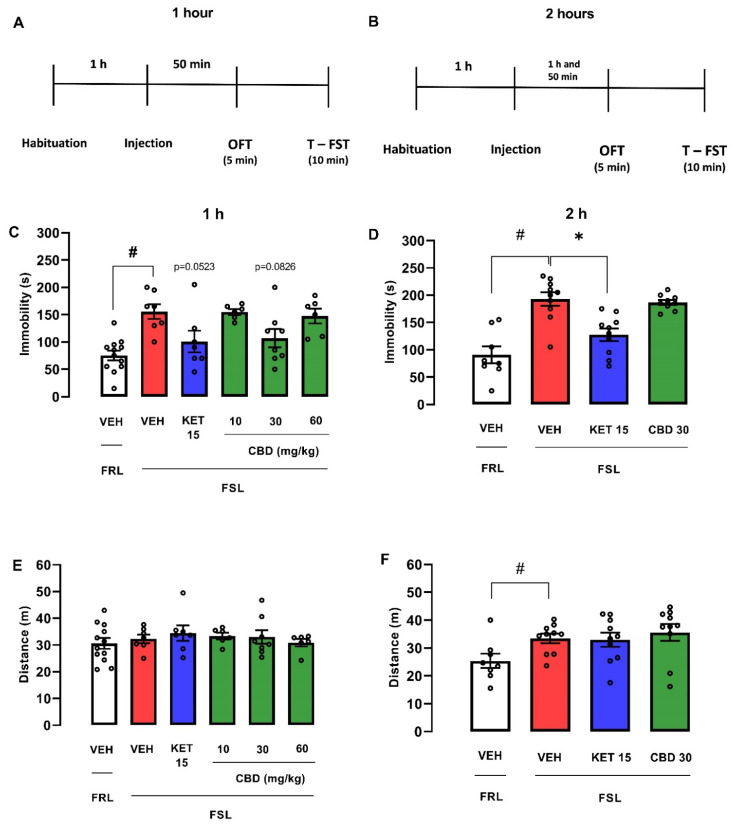
Effects of cannabidiol (CBD) administered 1 or 2 h before the forced swim and open field tests in male FSL rats. Experimental scheme (**A**,**B**). Effect of cannabidiol (CBD) in male FSL rats administered 1 (**C**,**E**) or 2 h (**D**,**F**) before the FST and OFT. Bars represent the immobility time (s) in FST or the traveled distance (m) in the OFT. Values are mean  ±  SEM; hash indicates significant differences between FSL and FRL vehicle-treated groups (# *p* < 0.05, Student’s *t*-test); asterisk represents significant treatment difference from FSL control (* *p* < 0.05; one-way ANOVA followed by Dunnett post hoc test or Kruskal–Wallis followed by Dunn’s post hoc), n = 6–12 animals/group. CBD: cannabidiol; FST: forced swimming test; OFT: open field test; KET: S-ketamine; VEH: vehicle.

One hour after injection, male FSL rats treated with vehicle displayed significantly increased immobility time compared with FRL rats treated with vehicle (Student’s *t*-test: t(17) = 5.126; *p* < 0.0001; Figure 6C). FSL rats treated with CBD (30 mg/kg) or KET (15 mg/kg) showed a tendency to reduce immobility time (one-way ANOVA: F(4, 29) = 3.178; *p* = 0.0279; Dunnett test: CBD 30 mg/kg, *p* = 0.0826; KET, *p* = 0.0523; Cohen d test: FSL-VEH vs. FSL-KET, d = 1.218; FSL-VEH vs. FSL-CBD 30 mg/kg, d = 1.153; Figure 6C), suggesting an antidepressant-like effect. Neither rat strain (t(17) = 0.5769; *p* = 0.5716) nor drug treatment (one-way ANOVA: F(4, 29) = 0.3576; *p* = 0.8366; Figure 6E) changed the locomotor activity in FSL rats.

Two hours after injection, male FRL treated with vehicle presented lower immobility (Student’s *t*-test: t(16) = 5.241; *p* < 0.0001; Figure 6D) and increased locomotion (Student’s *t*-test: t(16) = 2.722; *p* = 0.0151; Figure 6F) in comparison with vehicle-treated FSL animals. As demonstrated previously, ketamine (15 mg/kg) injected 1 h before FST reduced immobility in FSL rats (Kruskal–Wallis test: H(3) = 14.52; *p* = 0.0007; Dunn’s test: *p* = 0.0007; Cohen d test: d = 1.749; Figure 6D). CBD did not change immobility in the test 2 h later (Kruskal–Wallis test: H(3) = 14.52; *p* = 0.0007; Dunn’s: *p* > 0.9999; Figure 6D). None of the treatments changed the distance traveled in the OFT (one-way ANOVA: F(2, 27) = 0.3255; *p* = 0.7250; Figure 6F).

**Figure 7:**
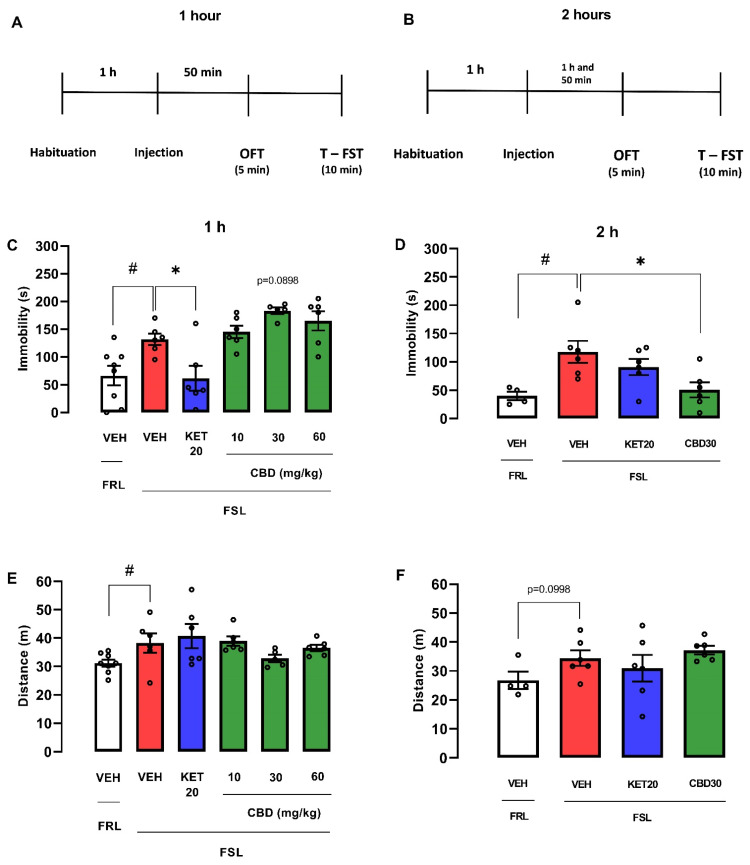
Effects of cannabidiol (CBD) administered 1 or 2 h before the forced swim and open field tests in female FSL rats. Experimental scheme (**A**,**B**). Effect of cannabidiol (CBD) in female FSL rats administered 1 (**C**,**E**) and 2 h (**D**,**F**) before the exposure to the FST and OFT. Bars represent the immobility time (s) in the FST or the traveled distance (m) in the OFT. Values are mean  ±  SEM; hash indicates significant differences between FSL and FRL vehicle-treated groups (# *p*  <  0.05, Student’s *t*-test or Mann–Whitney test); asterisk represent significant treatment difference from FSL control rats (* *p*  <  0.05; one-way ANOVA followed by Dunnett post hoc), n  =  4–10 animals/group. CBD: cannabidiol; FST: forced swimming test; OFT: open field test; KET: S-ketamine; VEH: vehicle.

On the other hand, female FSL rats treated with vehicle displayed significantly increased immobility time when compared with FRL rats treated with vehicle (Student’s *t*-test: t(12) = 2.954; *p* = 0.0120; Figure 7C). They also showed a significant decrease in locomotion (Mann–Whitney test: U = 8; *p* = 0.0426; Figure 7E). However, female FSL rats treated with the intermediate dose of CBD (30 mg/kg) showed a tendency to increase immobility in the FST 1 h after treatment (one-way ANOVA: F(4, 24) = 9.464; *p* < 0.0001; Dunnett test: *p* = 0.0898; Figure 7C), suggesting a depressive-like effect. In contrast, the FSL rats treated with ketamine (20 mg/kg) showed a significant reduction the immobility (one-way ANOVA: F(4, 24) = 9.464; *p* < 0.0001; Dunnett test: *p* = 0.0096; Figure 7C). No drug treatment in FSL rats affected the locomotor activity (Kruskal–Wallis test: H(5) = 5.917; *p* = 0.2054; Figure 7E).

Two hours after injection, female FSL rats treated with vehicle displayed significantly increased immobility time when compared with FRL rats treated with vehicle (Student’s *t*-test: (t(8) = 3.076; *p* = 0.0152; Figure 7D). They also presented a tendency to decrease the distance traveled in OFT (t(8) = 1.861; *p* = 0.0998; Figure 7D). Interestingly, 2 h after treatment CBD (30 mg/kg) in female FSL rats, immobility was reduced (one-way ANOVA: F(2, 15) = 4.439; *p* = 0.0306; Dunnett test: *p* = 0.018; Cohen d test: d = 1.621; Figure 7D). Ketamine (20 mg/kg) injected 2 h before the FST did not change the behavior in the test (one-way ANOVA: F(2, 15) = 4.439; *p* = 0.0306; Dunnett test: *p* = 0.4096; Figure 7D). No treatment modified the distance traveled in the OFT in FSL rats (one-way ANOVA: F(2, 15) = 0.9503; *p* = 0.4087; Figure 7F).

## 3. Discussion

The main finding of the present work was that CBD differentially modulates depressive-like behavior depending on injection time, strain, species, and sex. This study was the first to systematically investigate CBD’s behavioral effects in both sexes of FSL rats and different mice strains (Swiss and C57BL/6). Our main findings suggest that CBD produces an antidepressant-like effect in male Swiss mice but not in C57BL/6J mice. Strikingly, CBD induced a bimodal effect in female FSL rats depending on the injection time. In contrast, CBD tended to decrease the immobility time at 1 h after the injection in males and did not produce any behavioral change at 2 h.

Previous studies reported that acute injection of CBD produces an antidepressant-like effect in male Swiss mice subjected to different predictive tests, including FST [24,25,26,44] and TST [27], as we also observed herein. However, we failed to find an antidepressant effect of acute CBD treatment in male C57BL/6 mice in the TST. Previous studies reported that acute and repeated CBD administration produced a behavioral response in male C57BL/6 mice submitted to OBX [28] and CUMS [45,46], which suggests that CBD effects in C57BL/6J mice might depend on the experimental paradigm and treatment regimen (acute vs. repeated administration). Curiously, imipramine also did not induce an antidepressant-like effect in both male and female C57BL/6 mice in the TST, which contrasts with other evidence in the literature [47]. However, it is known that C57BL/6 mice are less sensitive than other strains of mice in the TST [38], and experimental conditions, such as age and previous stress exposure [48,49,50,51], can affect the results, which might explain the contrasting results between the studies. 

On the other hand, CBD did not change the immobility time in female Swiss and C57BL/6 mice submitted to the TST. Since CBD was tested in only one time point after drug administration (30 min) in mice, it is possible that other exposure times could produce different results in female mice, as we observed in female rats. Both pharmacokinetic and pharmacodynamic parameters might have influenced CBD effects in males and females of different mice strains. The peak of maximum plasma concentration (Cmax) of CBD can vary, depending on the route of administration, vehicle used, animal species, and sex [52]. Moreover, evidence has shown that mice strain (Swiss and C57BL/6) influences the baseline behavioral [38,40,53,54] response to established antidepressant drugs [37,38,40,49,55,56], neurochemical profile [37], sensitivity to stress [39,57,58], and some biochemical parameters [39], which could produce a significant impact on the CBD effects. Therefore, experimental differences might have contributed to the contrasting results between studies. 

The biological variable sex is a critical factor that affects the antidepressant drug response in different models [37,59,60,61,62,63] and can change the effective dose of a given drug [36,37,40]. For example, females may be more sensitive to the effects of selective serotonin reuptake inhibitors (SSRIs), since acute administration of lower doses of fluoxetine [59,61], paroxetine [37], and sertraline [64] produced an antidepressant-like effect in female rats and mice exposed to the FST and TST compared to males. Moreover, similar sex differences were reported in animals with the fast-acting antidepressant drug ketamine [60,65]. 

Surprisingly, we failed to detect CBD effects in mice submitted to the EPM. This contrasted with previous publications, where the anxiolytic properties of CBD were well described [27,45,46,54,66], but agrees with a recent publication in CD1 mice [67]. The lack of positive control in our EPM studies was a limitation for our conclusions about that. Anxiety disorders are frequently observed as a comorbidity in depressed patients [68,69]. However, in contrast to our expectations, CBD did not affect the anxiety-related behaviors in the EPM in male and female mice from both mice strains (Swiss and C57BL/6 mice). Previous works showed similar findings in male C57BL/6J mice, in which CBD acutely administered did not change the anxiety-related behaviors in different tests [53,54].

Moreover, consistent with our results in females, acute and repeated administration of CBD did not produce an anxiolytic effect in adolescent and adult female C57BL/6J mice [53]. However, in the OFT and light-dark test, CBD chronic administration did cause an anxiolytic effect after 21 days in male C57BL/6JArc mice [54]. It also prevented (after 14 days) the anxiogenic effect induced by chronic stress in the novelty suppressed feeding and EPM tests [45,46]. Until now, CBD effects on anxiety-related behaviors had not been investigated in female Swiss mice.

In male FSL rats, CBD showed a tendency to induce an antidepressant-like effect 1 h after the injection in the FST and did not produce any behavioral change 2 h post injection. A previous study from our group showed that CBD induced an antidepressant-like effect 1 h after administration but no sustained effect 7 days later in male FSL rats [24]. CBD effect was consistent with previous studies, which reported that CBD acute injection significantly decreased the immobility in the FST [23,70], reduced the number of failures, and increased the number of escapes in LH [24]. The small sample size used herein might have compromised the possibility of detecting a significant effect of CBD, although a trend was observed at the dose of 30 mg/kg.

Previous evidence demonstrated that CBD produced a hedonic and antidepressant-like effect in male FSL and WKY rats 2 h after oral administration, while it had no effect in female FSL [31,32]. In contrast, our results demonstrated a bimodal effect of CBD in female FSL rats, with marked antidepressant effect only at 2 h post injection. The discrepancy in the behavioral effects can be explained by the different behavioral tests used, vehicle, and the administration routes used (i.p. vs. oral). CBD is a highly lipophilic and poorly soluble molecule, making it difficult to ascertain the correct dose [14,71]. The vehicle and the administration routes can affect its pharmacokinetic profile, thus resulting in different plasma levels, Tmax and Cmax [52,72,73,74]. Measurements of CBD plasma levels in these two time points clarifying these results.

What could then be the neurobiological explanation behind the sex differences observed in our work? One possible explanation would be the physico-chemical properties of CBD. CBD has a chemical structure that confers high lipophilicity similar to delta-9-tetrahydrocannabinol (Δ9-THC) [14,71]. Therefore, it rapidly penetrates highly vascularized tissues in a short time, accumulates in fat tissue, and suffers redistribution, modifying the plasmatic concentration of the drug [75]. Consequently, this process can be affected by body weight and composition, which varies between sexes [75,76,77,78]. CBD presents a similar molecular structure to Δ9-THC [79,80,81], and the influence of sex on Δ9-THC metabolism in the rat liver has been previously shown [82,83]. Adult female rats presented higher blood levels of the hydroxylated metabolite, 11-hydroxy-Δ9-THC, than males [83]. Therefore, it is likely that these factors can influence the plasmatic and brain concentration of CBD and, consequently, differentially impact the behavioral effects observed among different sexes.

Another possible explanation for sex differences could be related to pharmacodynamic aspects. A growing body of evidence has shown sexual dimorphisms in the endocannabinoid and serotonergic systems, key molecular targets enrolled in the CBD effect [16]. For example, the gonadal hormones influence the levels of endocannabinoids [84] and serotonin [85], as well as cannabinoid receptor [86,87] and serotoninergic receptor type 1A (5-HT1A) expression [88,89]. Indeed, further studies are crucial to understanding the molecular mechanism involved in the CBD effects in females. 

The present study was also the first to perform a dose-effect curve for ketamine in female FSL rats. We showed that a single injection of ketamine (15 and 20 mg/kg) produced an antidepressant-like effect in female FSL rats exposed to FST. Our finding strengthened previous results showing the efficacy of the treatment with ketamine in female rats, even though the effective doses vary in comparison to males [90,91,92,93,94]. In agreement with these results, our group recently showed that ketamine responses in FSL rats were associated with a sex difference in the hippocampus morphology, alteration of hippocampal astrocytes, and brain-derived neurotrophic factor (BDNF) [95]. More studies are necessary to investigate how these differences are involved in the ketamine effect in female FSL rats.

Notwithstanding, there are a few limitations in the present work. Most importantly, it would be relevant to measure CBD plasma and brain levels in both sexes. It would also be interesting to consider the estrous cycle in females and how it may affect the basal response in the tests [96,97,98,99]. Even if it seemed not to modulate the FST response in female FSL rats [100], it could influence the drug effects of the other tested species. Due to the limitations of the TST as a model of depression [47,101], it would be interesting to investigate the effect of CBD using additional preclinical models. Finally, a positive control group in the EPM using an anxiolytic benzodiazepine could help to confirm the lack of CBD effect in anxiety-related behaviors in both sexes and strains of the used mice [102,103,104].

## 4. Materials and Methods

### 4.1. Animals

We used adult male and female Swiss and C57BL/6 mice (8 weeks old) from the University of São Paulo (SP) breeding facility and adult male and female Flinders Sensitive Line (FSL) and Flinders Resistant Line rats (FRL; control of genetic background) (weight: male: 200–405 g; female: 138–216 g; 8–12 weeks old) from breeding colonies at Translational Neuropsychiatry Unit (Aarhus University, Aarhus, Denmark). The mice were housed in groups of 10 animals per polypropylene cages (200 × 120 × 300 mm), and the rats were housed in pairs in standard cages (Cage 1291H Eurostandard Type III H, 425 × 266 × 185 mm, Tecniplast, Buguggiate, VA, Italy). All animals were housed in a temperature-controlled room (23 ± 2 °C) with a 12/12 h light-dark cycle (lights on 6:00 a.m./lights off 6:00 p.m.) with free access to tap water and standard food. The bedding material for rats (Tapvei Estonia OÜ, Paekna, Estonia) was made of wood chips with access to tunnel shelter, nesting material, and a wooden stick. For mice, cages were lined with wood shavings without enrichment material in the cages. Female and male animals were allocated in different rooms to avoid interference in the behavioral results. 

The Ethics Committee approved the experimental protocols for the use of animals from the School of Pharmaceutical Sciences of Ribeirão Preto—USP (protocol number 17.1.537.60.6) and the Danish Animal Experiments Inspectorate (protocol number 2016-150201-001105). The experimental procedures were conducted following the National Council for Control of Animal Experimentation (CONCEA, Brasília, DF, Brazil) and European Community Council Directive 2010/63/EU. All behavioral experiments were conducted between 9:00 a.m. and 1:00 p.m.

### 4.2. Drugs

Synthetic cannabidiol (CBD; Prati-Donaduzzi, Toledo, PR, Brazil; doses: 3, 10, and 30 mg/10 mL/kg intraperitoneal (i.p.)) stored at 4 °C and protected from light, diluted with sterile saline and 2% polysorbate 80 (Tween^®^ 80; Synth, Diadema, SP, Brazil) for mice [24,25,26] or synthetic CBD (THC-Pharma, Frankfurt, HE, Germany; doses: 10, 30, and 60 mg/kg/2 mL i.p.) diluted with sterile saline and 3% polysorbate 80 (Tween^®^ 80; Sigma-Aldrich, St. Louise, MO, USA) for systemic administration in FSL rats [24]. S-ketamine hydrochloride (Pfizer Ltd.a, Ballerup, Denmark; doses: 10, 15, and 20 mg/kg/2 mL i.p.) stored at 4 °C was dissolved in sterile saline [24,105] and imipramine hydrochloride (IMIP; Abcam, Waltham, MA, USA; dose: 20 mg/kg/10 mL), stored at 4 °C and diluted in sterile saline [106]. The vehicle (VEH) group received CBD vehicle injections. All drugs were freshly prepared before the experiment. The animals received the treatment randomly by writing down the treatment in pieces of paper, folding them, then drawing one by one for each animal [107].

### 4.3. Methods and Experimental Design

#### 4.3.1. Mice

##### Experiments 1 and 2—CBD Effects in Male and Female Mice Exposed to EPM and TST

The elevated plus maze (EPM) and the tail suspension test (TST) were conducted to investigate the anxiolytic and antidepressant-related behaviors in mice. The EPM was performed as previously described [27,108]. The apparatus was a plus-shaped maze made of wood and consisted of 2 equals enclosed arms (EA; 30 cm × 6 cm; surrounded by walls 15 cm high) disposed perpendicularly to a 2 equals open arms (OA; 30 cm × 6 cm). The animals were placed in the center of the equipment facing one EA and freely exploring the maze for 5 min. The OA entries, time, and percentage of time spent in the OA were analyzed. Also, EA entries were assessed as an exploratory behavior, as described in [103,108].

In the TST, the animal was suspended 60 cm above the floor with the adhesive tape placed 1 cm at the tip of the tail on the experimentation table for 6 min [47,109]. Moreover, a plastic cylinder tubing (40 mm length; 16 mm diameter) was placed around the animal tail to prevent tail-climbing behavior, as described previously [110].

Thirty minutes after the habituation period in the experimental room, the mice received the intraperitoneal injection with VEH, IMIP, or CBD (3, 10, and 30 mg/kg). Thirty minutes later, the animals were exposed to the EPM (5 min) and TST (6 min). To avoid interference in the behavioral response in the tests, the experiment carried out with females was performed independently and on different days from male animals. The female reproductive cycle status was not taken into account [111].

Independent experiments were carried out for Swiss and C57BL/6 mice for each sex (a total of four independent experiments).

#### 4.3.2. Flinders Sensitive Line (FSL) Rats

##### Experiment 3—CBD Effect 1 and 2 h before OFT/FST in Male FSL Rats

To assess whether CBD produced an antidepressant-like effect 1 or 2 h after the injection, the rats received an i.p. injection with VEH, ketamine (15 mg/kg), or CBD (10, 30, and 60 mg/kg). After 50 min or 1 h 50 min, the animals were exposed to the OFT (5 min) and, immediately after, they were submitted to the FST (5 min). The FST was performed, as previously described [24,112]. The FSL and FRL rats were exposed to a 10 min test in a Perspex cylinder (height 60 cm, diameter 24 cm) filled with tap water at 24 ± 1 °C, up to 40 cm height, and the immobility time was measured in the first 5 min [24].

To analyze the unspecific change in the locomotor activity produced by the drug treatment, an open field test (OFT) was performed, as previously described [113]. Immediately before the test in the FST (see below), the rats were submitted individually to an open field square (100 cm × 100 cm) for 5 min. The light intensity was 40 lux at the center of the arena. The total distance traveled (meter; m) was subsequently analyzed using EthoVision^®^ XT14 (Noldus Information Technology, Wageningen, The Netherlands).

In addition, the FSL and FRL rats were handled for 3–5 min during three consecutive days before the experiment to habituate the animals to the experimenter and minimize stress caused by manipulation.

##### Experiment 4—Dose-Response Curves with Ketamine in Female FSL Rats Exposed to OFT/FST

A dose-response curve with ketamine was conducted to determine the effective dose that produced antidepressant-like effects in female FSL rats. For this purpose, 1 h after the habituation in the experimental room, the female FRL rats received systemic treatment with VEH (sterile saline), and female FSL rats were treated with VEH or ketamine (10, 15, and 20 mg/kg i.p.). Fifty minutes later, the rats were submitted to OFT (5 min) and FST (10 min), as previously described. The rat reproductive cycle status was not considered, as it was earlier shown not to affect immobility [100,114]. 

##### Experiment 5—CBD Effect 1 and 2 h before OFT/FST in Female FSL Rats

A similar design described in Experiment 3 was used for females, except that S-ketamine was 20 mg/kg. To avoid interference in the behavioral response in the tests, the experiment carried out with females was conducted independently and on different days than the males. The rat reproductive cycle status was not considered [100,114].

### 4.4. Data Analysis

A three-way analysis of variance (ANOVA) with treatment, sex, strain or time (with FSL rats) as independent factors was performed to examine the behavioral data. To measure significant effects of the strain and in the interaction (treatment vs. strain), we performed an independent two-way ANOVA to evaluate the treatment and sex effect in each mice strain, Swiss and C57BL/6. In sequence, to measure significant effect on the treatment and interaction (sex vs. treatment), we performed a one-way ANOVA on each sex to compare the treatment effect on immobility. However, for FSL rats, to compare significant effects of the interaction (treatment vs. sex vs. time), we performed the following tests: (i) Student’s *t*-test to compare the results between the rat strain, FRL and FSL vehicle-treated groups and (ii) one-way ANOVA test followed by Dunnett post hoc test to compare differences between FSL-VEH and FSL-ketamine or FSL-CBD and mice treated groups (VEH, IMIP, and CBD). When the variances between the groups were not homogenous, Mann–Whitney (for comparisons between FSL and FRL vehicle-treated groups) or Kruskal–Wallis followed by Dunn’s post hoc test (to compare between FSL treated with VEH, ketamine, and CBD and treated mice) was applied. Significant outliers were removed from the statistical analysis through GraphPad’ Outlier calculator (online version, 2021; GraphPad Software Inc., San Diego, CA, USA). The outliers were the following: male and female FSL rats: 1 rat/sex; female C57BL/6 and male Swiss mice: 5 animals/strain; male C57BL/6 and female Swiss mice: 3 animals/strain. We calculated and reported the effect size from TST and FST using G*Power (version 3.1.9.6, Heinrich Heine Universität, Düsseldorf, NRW, Germany) [115]. Results in the graphs are expressed as the mean  ±  standard error of the mean (SEM). A significant difference between groups was considered when *p* ≤ 0.05. The *p*-value between 0.05 and 0.1 was considered a statistical trend [116]. Statistical analyses were performed using GraphPad Prism 8.0 (GraphPad Software Inc., San Diego, CA, USA) and SPSS software (version 20.0), and the graphs were created using GraphPad Prism 8.0 (GraphPad Software Inc., San Diego, CA, USA). The raw data are published on FigShare [117].

## 5. Conclusions

In conclusion, our findings point out that sex, animal strain, species, and injection time may affect the behavioral response induced by CBD in rodents submitted to animal models of depression. CBD produced an antidepressant-like effect only in male Swiss mice and no effect in female Swiss mice or C57BL/6 mice (both sexes) was observed. Notably, in female FSL rats, CBD produced an antidepressant effect 2 h post injection and tended to induce a depressive-like effect at 1 h. In male FSL, CBD tended to cause an antidepressant at 1 h but not at 2 h. Therefore, these findings indicate that it is necessary to consider the sex, animal strain and species, compound chemistry, exposure, and behavioral test when evaluating novel drugs for depression.

## Data Availability

The data presented in this study are openly available in FigShare at [https://doi.org/10.6084/m9.figshare.16583408.v1; https://doi.org/10.6084/m9.figshare.16583402.v1; https://doi.org/10.6084/m9.figshare.16583408.v1; https://doi.org/10.6084/m9.figshare.16583417.v1; https://doi.org/10.6084/m9.figshare.16589687.v1; https://doi.org/10.6084/m9.figshare.16589696.v1], reference number [117].
